# Effect of Five Bench Inclinations on the Electromyographic Activity of the Pectoralis Major, Anterior Deltoid, and Triceps Brachii during the Bench Press Exercise

**DOI:** 10.3390/ijerph17197339

**Published:** 2020-10-08

**Authors:** David Rodríguez-Ridao, José A. Antequera-Vique, Isabel Martín-Fuentes, José M. Muyor

**Affiliations:** 1 Health Research Centre, University of Almería, 04120 Almería, Spain; daviidrr89@gmail.com (D.R.-R.); josealmeria_591@hotmail.com (J.A.A.-V.); imf902@ual.es (I.M.-F.); 2 Laboratory of Kinesiology, Biomechanics and Ergonomics (KIBIOMER Lab.), Research Central Services, University of Almería, 04120 Almería, Spain

**Keywords:** EMG, chest press, muscle activity, resistance exercise, strength, fitness

## Abstract

The bench press exercise is one of the most used for training and for evaluating upper-body strength. The aim of the current study was to evaluate the electromyographic (EMG) activity levels of the pectoralis major (PM) in its three portions (upper portion, PMUP, middle portion, PMMP, and lower portion, PMLP), the anterior deltoid (AD), and the triceps brachii (TB) medial head during the bench press exercise at five bench angles (0°, 15°, 30°, 45°, and 60°). Thirty trained adults participated in the study. The EMG activity of the muscles was recorded at the aforementioned inclinations at 60% of one-repetition maximum (1RM). The results showed that the maximal EMG activity for PMUP occurred at a bench inclination of 30°. PMMP and PMLP showed higher EMG activity at a 0° bench inclination. AD had the highest EMG activity at 60°. TB showed similar EMG activities at all bench inclinations. In conclusion, the horizontal bench press produces similar electromyographic activities for the pectoralis major and the anterior deltoid. An inclination of 30° produces greater activation of the upper portion of the pectoralis major. Inclinations greater than 45° produce significantly higher activation of the anterior deltoid and decrease the muscular performance of the pectoralis major.

## 1. Introduction

The upper body is constantly involved in daily athletic activities such as push and throwing events. The muscles most involved in these activities are the pectoralis major, the anterior deltoid, and the triceps brachii. The pectoralis major is a large muscle consisting of three portions (clavicular, sternal, and costal portions). Altogether, this muscle acts as a powerful medial rotator and adductor of the arm. The anterior deltoid muscle assists the pectoralis major to flex the shoulder, while the triceps brachii is the primary elbow extensor.

Accordingly, the bench press exercise is one of the most used for upper-body resistance training by both athletes and recreational trainers [1] to improve their performance or as a measure of upper-body strength [2]. Moreover, competitive bench press athletes use numerous variations of the bench press to isolate and train the shoulder girdle muscles, which contribute significantly to performance [3]. In fact, the barbell bench press is considered the best exercise for pectoral development [4]. For this, the bench inclination is one of the most used variables for modifying muscle activity during the bench press exercise. Specifically, by modifying the bench angle during the bench press exercise, the greatest muscle activation of either the upper or the lower pectoralis major can be elicited [5].

Several studies have evaluated the electromyographic (EMG) activity of the pectoralis major by varying the bench press inclination (incline and decline bench press) [3,5,6,7,8]. Barnett et al. [6] investigated how the EMG activity of five muscles acting at the shoulder joint was affected by varying the bench inclination and hand spacing. These authors found that the sternocostal head of the pectoralis major was more active when using the horizontal bench press than the incline bench press. However, these authors considered the sternal and costal portions of the pectoralis major as a single portion. Moreover, in this study, over the horizontal plane, the authors only compared three bench press inclinations (horizontal, 40° above horizontal, and the vertical “military press”) [6]. Glass and Armstrong [7] compared the EMG activity of the upper pectoral (clavicular portion) and the lower pectoral (costal portion) at two bench press inclinations (+30° and −15° from horizontal). Their results showed significantly greater lower pectoral activation with a decline bench press, and similar activation of the upper pectoral was found with incline and decline bench press. However, the authors did not evaluate the EMG activity of the other muscles engaged by the bench press such as the middle pectoral (sternal portion), anterior deltoid, or triceps brachii nor did they evaluate any other bench press inclinations. Trebs et al. [8] compared the clavicular head and sternocostal head activation of the pectoralis major and the anterior deltoid when performing the bench press at 0°, 28°, 44°, and 56° above horizontal using 70% of one-repetition maximum (1RM) for each angle. These authors found that the clavicular heads of the pectoralis major and the anterior deltoid showed greater muscle activation as the bench press’s inclination was increased. However, like Barnett et al. [6], these authors considered the sternal and costal portions of the pectoralis major as a single portion and they did not evaluate other synergist muscles such as the triceps brachii. Similarly, Lauver et al. [5] compared the muscular activation of the upper and lower pectoralis major, anterior deltoid, and triceps brachii during a free-weight barbell bench press performed at −15°, 0°, 30°, and 45° bench angles. They reported that the horizontal bench press could be used to achieve muscular activation of both the upper and the lower heads of the pectoralis. However, these authors did not analyze the middle portion of the pectoralis major. More recently, Saeterbakken et al. [3] compared EMG activity while performing a 6RM competition style bench press on a flat (0°), incline (+25°), and decline (−25°) bench. Their results reported non-significant differences in activation for the three bench positions, except for a lower activation of the triceps brachii and a greater activation of the biceps brachii with the incline bench press than with the horizontal and decline bench press. Although these authors also analyzed the EMG activity with respect to grip widths for these bench press inclinations, they only measured a single bench press inclination angle (+25° from horizontal).

Previous studies demonstrated that the decline bench press activates the lower pectoralis major [3,5,7], the flat bench press activates the sternocostal pectoralis major, and the incline bench press activates the upper pectoralis major [6,8]. For this reason, it is commonly believed that the horizontal bench press exercise is effective for stimulating the middle portion of the pectoralis major, and when the bench press is inclined above horizontal, the EMG activity produced in the upper portion of the pectoralis major is greater.

Although the above statements seem straightforward, Glass and Armstrong [7] reported no significant differences in the upper pectoral major with the incline and the decline bench press. Lauver et al. [5] concluded that a decline or a 45° bench incline would have little or no added benefit in improving muscle strength and developing the pectoralis major. Moreover, we know of no studies that have evaluated the EMG activity of the three pectoralis major portions to determine the bench press inclination angle associated with greater EMG activity of the pectoralis major upper portion, or when the anterior deltoid starts to be highly activated at these bench inclinations with the participation of the triceps brachii. Recently, Muyor et al. [9] analyzed the three portions of the pectoralis major, the anterior deltoid, and the triceps brachii, among other muscles, during the bench press exercise. However, these authors only compared the EMG activity using the horizontal bench press when the athletes had their feet on the ground and with an active hip flexion, but not at different bench press inclinations.

Therefore, the aim of the current study was to evaluate the levels of EMG activity in the three portions (clavicular, sternal, and costal) of the pectoralis major as well as in the anterior deltoid and the triceps brachii (medial head) during the bench press exercise at five bench angles (0°, 15°, 30°, 45°, and 60°).

## 2. Materials and Methods

### 2.1. Participants

Thirty young, healthy, and physically active adults participated voluntarily in the study. They had a minimum of 1 year of resistance training experience, performing at least three sessions per week from moderate- to high-intensity resistance training exercises. The characteristics of the sample group are shown in Table 1.

The participants did not present any injury or limitation hindering the performance of the required exercises. Furthermore, they had no musculoskeletal injuries in the 12 months after the completion of this study.

The University of Almería’s institutional review board approved this study, and all participants were informed about the objectives and procedures of this study before providing written informed consent. All procedures were conducted according to the Declaration of Helsinki.

### 2.2. Procedures

Each participant visited the laboratory on two occasions, set apart by a minimum of 48 h to avoid the effects of muscle fatigue [10]. For each visit, the subjects were instructed to avoid strenuous exercise involving the upper body for 48 h before testing. During the first visit, the participants determined the load to be lifted in a maximum repetition (1RM) of the bench press exercise at each bench inclination. The second test session was used to record the electromyographic activity of the muscles in order to evaluate the bench press exercise at each of the five inclinations (0°, 15°, 30°, 45°, and 60°). The participants performed the lifts at each inclination in a randomized order to minimize any order effect.

#### 2.2.1. Determination of the 1RM

The first session began by taking measurements of each participant—their biacromial width, body mass (using an electronic body composition analyzer (model BF−350; Tanita, Tokyo, Japan)), and height (using a Seca stadiometer (Seca, Hamburg, Germany))—as reported in Table 1.

Then, the participants performed a standardized warm-up on an elliptical machine (to mobilize the upper extremities). Next, the participants performed dynamic active stretching and joint mobility exercises for 5 min, for the body segments involved in the bench press exercises. In no case did the warm-up cause fatigue in the participants.

Then, the participants performed a specific protocol on the bench press to reach the 1RM [11,12] for each inclination. They performed from 4 to 5 sets for each bench press inclination: (1) 20 repetitions at approximately 30% 1RM, (2) 12 repetitions at approximately 50% 1RM, (3) 6 repetitions at approximately 70% 1RM, and (4) 1 repetition at approximately 85% 1RM. They were able to increase the minimum weight of 8 kg during the 1RM test, which corresponded to 85%–100% 1RM at 60°. Finally, the participants had to lift the maximum weight possible in a single repetition (considered as 1RM) while maintaining an adequate technique [13]. Usually, in this way, the 1RM was established at the second attempt. However, if necessary, a third attempt was made. The rest periods between sets and between bench press inclinations were from three to five minutes to avoid muscle fatigue. None of the participants showed symptoms of fatigue that limited the performance or reliability of the tests. The 1RM for each bench press inclination was calculated in the morning and in a randomized and counterbalanced order.

#### 2.2.2. Electromyography Setup and Data Collection

The protocol was the same as that used by Muyor et al. [9]. It started with the same cardiovascular warm-up—dynamic active stretching and joint mobility exercises—as in session 1.

Next, the areas chosen for electrode placement were prepared by shaving the off hair and cleansing with alcohol to reduce surface impedance. Ag/AgCl electrodes (Medico Lead-Lok, Noida, India) were placed parallel to the muscle fibers at a 2 cm center-to-center distance.

**Figure 1:**
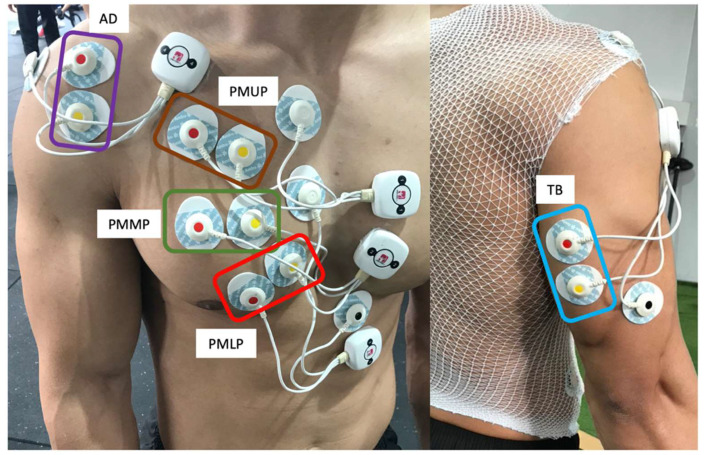
Electrode placement diagram for recording electromyographic (EMG) activity. AD: anterior deltoid; PMUP: pectoralis major upper portion, clavicular portion; PMMP: pectoralis major middle portion, sternal portion; PMLP: pectoralis major lower portion, costal portion; TB: triceps brachii, medial head.

Following the Surface Electromyography for the Non-invasive Assessment of Muscles (SENIAM) recommendations [14], the electrodes were placed on the dominant side of each participants and were fixed with an adhesive tape to prevent possible displacement during the exercises. Specifically, the electrodes were placed as follows: on the pectoralis major upper portion (clavicular portion, PMUP), at the midclavicular line over the second intercostal space [7]; on the pectoralis major middle portion (sternal portion, PMMP) of the chest wall, horizontal to the rising muscle mass (approximately 2 cm from the axillary fold) [15]; on the pectoralis major lower portion (costal portion, PMLP) at the midclavicular line over the fifth intercostal space [7]; on the anterior deltoid (AD) at 1.5 cm distal and anterior to the acromion [1] and on the triceps brachii (TB) medial head at the midpoint between the posterior aspect of the acromion and the olecranon processes [16] (Figure 1).

After placing the electrodes, the maximal voluntary isometric contraction (MVIC) of each muscle was recorded to normalize the EMG values registered at each bench press inclination. To do that, two 3 s MVICs trials were recorded for each muscle in a randomized manner, with approximately a 10 s rest interval between each contraction and 2 min between the MVIC measure of each muscle [17]. The MVIC was determined as an average amplitude over a one-second window of the highest rectified EMG signals (root-mean-square, RMS) with a 100 ms window [18,19,20].

The methodology used for the MVIC has previously been described [9]. Specifically, the MVIC was determined as follows: for the pectoralis major (upper, middle, and lower portions), the participants performed an isometric bench press with a grip at 150% of biacromial width, the shoulder abducted at 45°, and the feet flat on the bench. For the anterior deltoid, the participants performed a deltoid flexion at 90° in a seated position, an erect posture with no back support. For the triceps brachii, the participants performed a forearm extension with elbows at 90° in a seated position, an erect posture with no back support. In all cases, with the exception of the isometric bench press exercise for the pectoralis major, which was performed with a maximum load, a maximum manual resistance was opposed by a researcher in the opposite direction to the muscular motion. All the muscles were randomly tested to avoid fatigue. To maintain a consistent effort during the MVIC, an examiner provided verbal encouragement to each participant.

The EMG activity of the MVICs demonstrated high reliability, with intraclass correlation coefficient (ICCs) ≥0.9 and coefficient of variation (CV) ≤3%.

Subsequently, each participant performed a more specific warm-up, consisting of 20 repetitions at 30% 1RM on the incline of the bench where the test would begin. Finally, after a 5 min rest, bench press data were collected for each bench press inclination (0°, 15°, 30°, 45°, and 60°) in a random and counterbalanced order with a 5 min rest between trials. The bench angles were measured using a Uni-Level inclinometer (ISOMED, Inc., Portland, OR, USA). The participants performed a set of 8 repetitions at 60% 1RM, at a rate of two seconds for the eccentric phase and two seconds for the concentric phase [21,22] for each bench press inclination. The velocity of the reps was controlled by a KORG MA−1 metronome (Keio Electronic Laboratories, Tokyo, Japan).

Regarding the technique during the exercises, all participants started the exercise lying in the supine position on the bench, gripping the bar (using a hook grip with the thumb) at 150% of the biacromial width and with the hands and forearms pronated during all repetitions. The bar was lowered to 1 cm from the chest (sternum), with the shoulders abducted to approximately 45° (in the eccentric phase), and the bar was raised until the elbows were extended (avoiding elbow hyperextension) to stay in the start position (in the concentric phase). During the whole exercise, the participant’s head and back (thoracic area) were kept in contact with the bench to prevent any inertial motion, and the bar was kept entirely perpendicular to the sternum (at the height of the nipples). This bench press procedure was maintained for all inclinations.

### 2.3. Electromyography

Electromyography signals for each muscle were recorded using a WBA Mega device (Mega Electronics Ltd., Kuopio, Finland) and were sampled at 1000 Hz. The analog signal was converted to a digital one via an A/D converter (National Instruments, New South Wales, Australia) and filtered by bandwidth (12–450 Hz) using a fourth-order Butterworth filter with the LabView software program (National Instruments, Austin, TX, USA). The raw EMG signals were then converted into RMS signals in microvolts (µV) with the MEGAWIN software program (Mega Electronics Ltd., Kuopio, Finland) for further analysis.

During the exercises, an electrogoniometer (Biometrics Ltd., Newport, UK) fixed on the lateral side of the ulna and humerus was used for identifiying each repetition. In this sense, each contraction was determined as the EMG signal between the start and the end points determined by the electrogoniometer traces, without differentiating concentric and eccentric phases.

**Figure 2:**
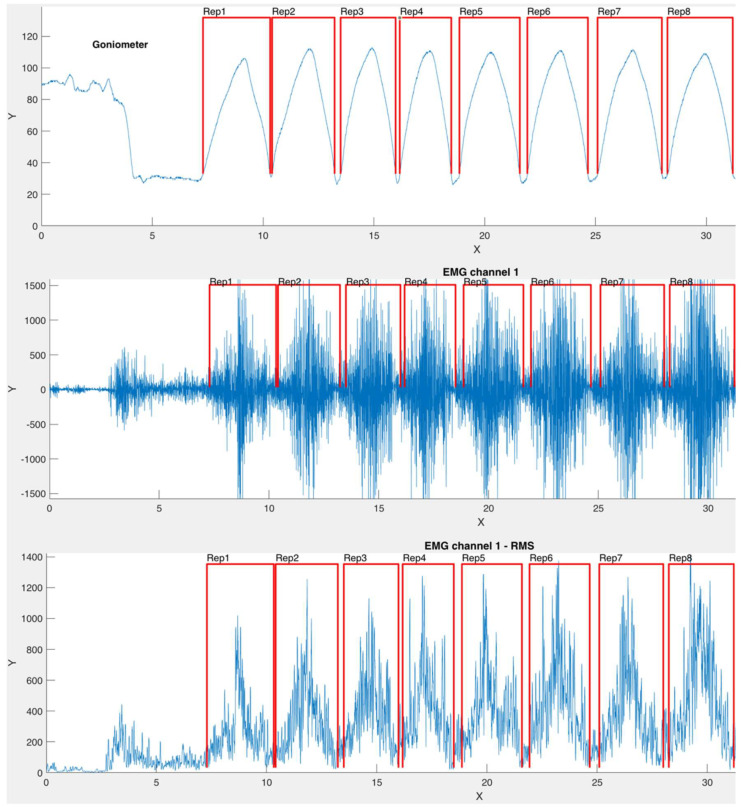
Example of the angular values and EMG tracing from a participant during the bench press exercise at 0°. The first to the third rows represent the angular values of elbow flexion–extension, raw EMG, and root-mean-square (RMS) EMG of the pectoralis major (sternal portion), respectively.

Although data were collected during eight repetitions, the first (initial) and the last one (8th) were removed for further analysis. The data from the electrogoniometer were continuously recorded and synchronized with the EMG data using the EMG equipment (Mega Electronics Ltd., Kuopio, Finland). A custom algorithm was developed in Matlab v. R2020a (Mathworks Inc., Natick, MA, USA) to automate the detection of each repetition (Figure 2).

### 2.4. Statistical Analyses

The Shapiro–Wilk normality test was used for analyzing the data distribution. Parametric tests were performed because all variables followed a normal distribution.

An analysis of variance (ANOVA) with a 5 × 5 (bench press inclination*muscle) design was performed to determine the differences in the EMG activity (% MVIC) for each muscle over the five bench press inclinations and to determine differences in the EMG activity (% MVIC) between the five muscles in each bench press inclination.

In addition, we performed the Mauchly´s sphericity test using all the ANOVA results to assess the assumptions of variance. In an attempt to reduce the type I error of the repeated measures ANOVA, the degrees of freedom were corrected using the Greenhouse–Geisser method when sphericity assumptions were not upheld. Bonferroni adjustment was employed, in pairwise comparisons, if a significant main effect was observed.

The sample size and statistical power were calculated with the software program G *power 3.1 for Mac OS X (Apple Inc., Cupertino, CA, USA) [23]. The statistical power was >0.9 for all the variables analyzed with the sample size used in the current study. Statistical analyses were carried out using the IBM SPSS software v.26 (IBM, Armonk, NY, USA), and the level of significance was set at alpha of 0.05 (*p* < 0.05).

## 3. Results

**Figure 3:**
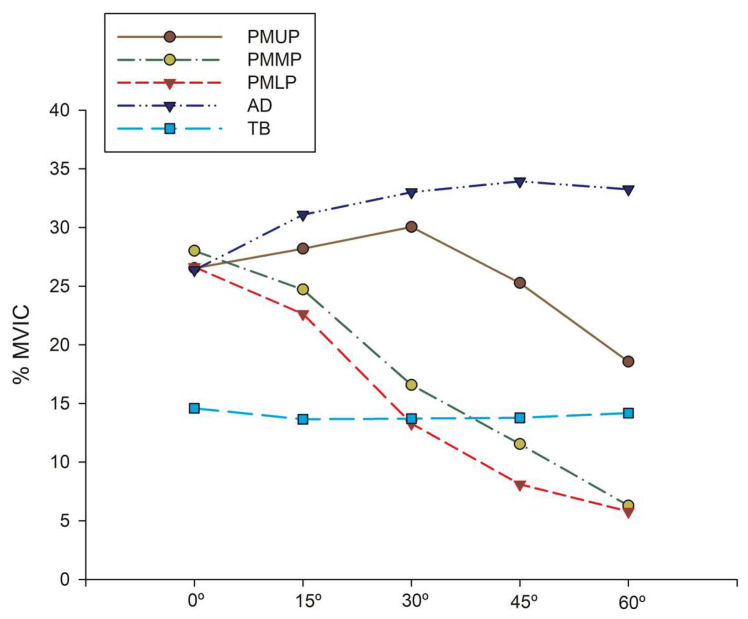
EMG activity (% maximal voluntary isometric contraction (MVIC)) of each muscle in relation to different bench press inclinations.

Regarding the normalized EMG activity for each bench inclination (Figure 3), the ANOVA showed that the PMMUP, PMMP, PMLP (~27% MVIC), and AD (~26% MVIC) did not present significant statistical differences at 0° (*p* > 0.05). The PMUP presented increased EMG activity at the 15° and 30° bench inclinations (~28% MVIC and ~30% MVIC, respectively) (*p* > 0.05). At 30°, the PMUP and AD presented similar EMG activity (~30% MVIC and ~33% MVIC, respectively) and higher EMG activity (*p* ≤ 0.01) than the rest of the muscles. The PMMP and PMLP presented lower EMG activity, with significant statistical differences when the bench inclination was increased (*p* ≤ 0.01). However, the AD presented greater EMG activity (~33% MVIC) when the bench was inclined, with statistically significant values at 45° and 60° bench inclinations (*p* ≤ 0.001) compared to the other muscles. The TB was equally activated at all bench inclinations (~15% MVIC) (*p* > 0.05), as shown in Figure 2 and Table 2.

## 4. Discussion

The primary aim of the current study was to evaluate the EMG activity levels in the three portions (clavicular, sternal, and costal) of the pectoralis major, the anterior deltoid, and the triceps brachii (medial head) muscles during the bench press exercise at five bench angles (0°, 15°, 30°, 45°, and 60°).

One of the main findings of this study was the modification of EGM activity of the pectoralis major (in its three portion) and AD when the bench press’s inclination was increased. These results agree with previous studies [5,6,7,8]. Barnett et al. [6] reported that, although the clavicular head of the pectoralis major showed greater muscle activation with the inclined bench press (40°), it was not statistically significant compared to the value measured with the horizontal bench press. However, Trebs et al. [8] found that the clavicular head of the pectoralis major experienced significantly heightened activity levels at bench angles of 44° and 56° but not at 28° (above horizontal); this contrasts with the finding of the current study. In our study, when we compared the EMG activity of the upper portion of the pectoralis major at bench press inclinations of 0° and 45°, we did not find any significant differences. Thus, the highest EMG activity presented for the clavicular head was at the 30° bench press inclination, and from that angle on, the AD presented the highest EMG activity (at 45° and 60° bench inclination). We agree with Lauver et al. [5], who reported that these differences might be explained by Trebs et al. [8] using a Smith machine. In our study, like in that performed by Lauver et al. [5], we used a free-weight barbell to conduct the bench press exercise.

Other notable findings were the higher EMG activity of the anterior deltoid as the bench press inclination increased, which is in agreement with [5,8], and a decrease in EMG activity of the lower pectoralis major [5,6,7,8]. According to Barnett et al. [6], this could be because the horizontal flexion and adduction requirements are minimal with the incline or vertical press. For this reason, the clavicular head of the pectoral major is less activated. However, Glass et al. [7] did not find a significant difference in the activation of the upper pectoral portion with either the incline (+30° from the horizontal plane) or the decline bench press (−15° from the horizontal plane). Nevertheless, they did not evaluate the EMG activity of other muscles, such as the middle portion of the pectoral, nor did they evaluate the EMG at other intermediate bench press inclinations, for example, with the bench press on the horizontal plane (0°). These authors did conclude that the incline bench press appeared to work well for stimulating EMG activity in the upper pectoral but not in the lower pectoral [7].

In the current study, the triceps brachii (medial head) maintained similar EMG activity at all the bench press inclinations (approximately 15% MVIC), presenting the lowest EMG activity compared to the other muscles evaluated, regardless of the bench incline used; this demonstrates the synergistic action of this muscle in the bench press exercise. However, previous studies found differences in EMG activity for the triceps brachii in relation to the bench press inclination [3,5,6]. Barnett et al. [6] found significantly less triceps brachii activity for both the incline and the vertical bench press compared to the horizontal one. However, these authors used different hand spacings (a biacromial diameter of 100% or 200%). This variable might affect the shoulder and elbow motion, influencing the EMG activity of the triceps brachii.

Another important finding of the current study is that the PMUP, PMMP, PMLP, and AD showed similar EMG activity at 0° bench inclination. These results are in agreement with previous studies [2,8,24]. Schick et al. [2] found no significant differences in muscle activation for the pectoralis major and anterior deltoid with the horizontal bench press. These authors only found significant differences in muscle activation for the medial deltoid when comparing the bench press exercise performed using a horizontal Smith machine with that performed using free weights; this is because the medial deltoid acts as a stabilizer muscle. As in our study, Trebs et al. [8] found that the sternocostal head of the pectoralis major was activated to a greater extent when the bench position was horizontal. Also, Gołaś et al. [24] recently observed similar muscle activity for the pectoralis major, anterior deltoid, and lateral head of triceps brachii in male athletes lifting a load of 55% 1RM. However, this muscle activity varied with other relative loads.

In accordance with previous studies [3,6,7,8], the same relative load (60% 1RM) was lifted at each bench angle to eliminate variations in the EMG activity caused by increasing the force required. As observed in this study, the absolute load lifted was lower as the bench angle increased. This result is in line with previous studies [6,7]. If the same load had been maintained for all bench press inclines, as in Lauver et al. [13], our EMG activity results would possibly have changed, given the modified relative load and force requirements.

The current work has certain limitations that could be tackled in future studies, such as controlled velocity during the repetitions to 2:2 to obtain a cleaner EMG signal [21,22]. For further studies, it may be interesting to measure this EMG activity in explosive executions or at high velocities, since the velocity of execution is considered an important variable of resistance training [25,26]. In addition, our study only assessed the EMG activity at a single relative intensity (60% 1RM). Future studies should analyze EMG activity at higher relative loads.

## 5. Conclusions

This study confirms that the bench press incline angle influences the EMG activity of different portions of the pectoralis major and the anterior deltoid. The horizontal bench press produces homogeneous electromyographic activity in the three portions of the pectoralis major and the anterior deltoid. In contrast, a 30° inclination produces greater activation of the upper portion of the pectoralis major. For inclinations above 30°, there is significantly greater activation of the anterior deltoid, which significantly decreases the EMG activity in the three portions of the pectoralis major.

## 6. Practical Applications

The horizontal bench press is a recommended exercise for maintaining or improving the dynamic performance of the upper body. Using more than a 45° bench incline is recommended to increase the performance of the anterior deltoid. The high activation of the anterior deltoid muscles during the bench press exercise is a variable to consider in the programming of strength training, due to the possible involvement of this muscle as a synergist muscle or its possible fatigue in scapulohumeral flexion exercises.

## Figures and Tables

**Table 1:** Descriptive characteristics of the sample group. Mean ± standard deviation. BMI: body mass index, 1RM: one-repetition maximum.

Variables	Mean ± SD
Age (years)	22.9 ± 3.0
Body mass (kg)	76.7 ± 8.7
Height (m)	1.8 ± 0.0
BMI (kg·m^−2^)	23.8 ± 2.1
150% biacromial width (cm)	68.3 ± 3.9
1RM Bench press 0° inclination (kg)	81.4 ± 15.5
1RM Bench press 15° inclination	72.0 ± 14.0
1RM Bench press 30° inclination (kg)	63.3 ± 12.3
1RM Bench press 45° inclination (kg)	57.9 ± 9.7
1RM Bench press 60° inclination (kg)	52.2 ± 9.0

**Table 2:** Pairwise comparisons between the EMG activity (relative to % MVIC) of each muscle and the bench press inclination.

Muscles	Bench Press Inclinations	15°	30°	45°	60°
PMUP	0°	0.977	0.688	1.000	0.002
	15°	–	1.000	0.559	0.000
	30°	–	–	0.214	0.000
	45°	–	–	–	0.000
PMMP	0°	0.004	0.000	0.000	0.004
	15°	–	0.000	0.000	0.000
	30°	–	–	0.000	0.000
	45°	–	–	–	0.000
PMLP	0°	0.005	0.000	0.000	0.000
	15°	–	0.000	0.000	0.000
	30°	–	–	0.000	0.000
	45°	–	–	–	0.016
AD	0°	0.170	0.014	0.001	0.000
	15°	–	1.000	0.028	0.032
	30°	–	–	0.116	0.185
	45°	–	–	–	1.000
TB	0°	1.000	1.000	1.000	1.000
	15°	–	1.000	1.000	1.000
	30°	–	–	1.000	1.000
	45°	–	–	–	1.000
